# Intestinal Epithelial-Protective Effects of *Leuconostoc mesenteroides* H40 in LPS-Stimulated HT-29 Cells

**DOI:** 10.4014/jmb.2607.07028

**Published:** 2026-09-04

**Authors:** Yu-Jin Cha, Hye Ji Jang, Gil-Woong Lee, Na-Kyoung Lee, Hyun-Dong Paik

**Affiliations:** 1 GHBio Co., Ltd., Seoul 05029, Republic of Korea; 2 Department of Food Science and Biotechnology of Animal Resources, Konkuk University, Seoul 05029, Republic of Korea; 3 Department of Bio-food and Foodservice Industry, Shinhan University, Gyeonggi-do 11644, Republic of Korea

**Keywords:** Probiotics, *Leuconostoc mesenteroides*, Intestinal inflammation, Intestinal barrier, Serotonin

## Abstract

In this study, the intestinal epithelial-protective effects of *Leuconostoc mesenteroides* H40 (H40) were investigated using HT-29 intestinal epithelial cells. H40 reduced nitric oxide production in sodium nitroprusside (SNP)-treated cells and suppressed the lipopolysaccharide (LPS)-induced gene expression of inflammatory mediators, including *interleukin-1 beta* (*IL-1β*), *IL-6*, *IL-8*, *TNF-α*, and *COX-2*. In addition, it attenuated the LPS-induced decrease in intestinal barrier-related genes, including *ZO-1*, *claudin-1*, *occludin*, *JAM1*, and *MUC2*, which was further supported by preserved or enhanced protein levels of claudin-1, occludin, and MUC2. Furthermore, H40 attenuated the LPS-induced reductions in TPH1 and SERT expression at both the mRNA and protein levels, suggesting a potential influence on serotonin-related molecular pathways in intestinal epithelial cells. Genome annotation revealed that H40 harbors genes associated with exopolysaccharide biosynthesis, cell wall/membrane-associated functions, cell-surface anchoring, antioxidant defense, tryptophan and aromatic amino acid metabolism, and B-vitamin metabolism, indicating that these genomic features may support its intestinal epithelial-protective potential and gut–brain axis-related functionality. Collectively, these findings revealed that H40 exerts anti-inflammatory, intestinal epithelial-protective effects, accompanied by modulation of intestinal barrier- and serotonin-related markers. These results indicate that H40 may represent a promising probiotic candidate for supporting intestinal epithelial health.

## Introduction

Evolving dietary patterns and increasingly irregular lifestyles have heightened interest in preserving intestinal health [1, 2]. The human gastrointestinal tract harbors a diverse microbial community that contributes to host health by regulating immune and metabolic processes, fermenting dietary components, and protecting against invading pathogens [3]. Therefore, maintaining microbial equilibrium is essential for suppressing potentially harmful microorganisms, controlling excessive inflammatory reactions, and supporting the structural and functional integrity of the intestinal epithelium [4]. Conversely, disruption of the intestinal microbial ecosystem can impair these protective mechanisms and has been implicated in the onset and progression of gastrointestinal diseases such as irritable bowel syndrome (IBS) and inflammatory bowel disease (IBD) [5]. Beyond its localized effects within the gut, the microbiota communicates with the central nervous system through the bidirectional microbiota–gut–brain axis, which involves neural, immunological, hormonal, and metabolite-mediated signaling pathways [6, 7]. Through this network, intestinal microorganisms can influence gastrointestinal function, stress-related responses, serotonergic balance, and emotional regulation. Accordingly, dysregulated microbiota–gut–brain communication may contribute to altered bowel motility, visceral hypersensitivity, and mood-related symptoms–key features in the pathophysiology of gastrointestinal disorders, particularly IBS [8, 9].

IBS is classified as a functional gastrointestinal disorder in which recurrent abdominal pain and altered defecation occur without detectable structural abnormalities [10]. In contrast, IBD, encompassing Crohn’s disease (CD) and ulcerative colitis (UC) involves chronic and relapsing intestinal inflammation accompanied by immune-mediated tissue damage [11]. Despite their distinct pathological and clinical characteristics, both disorders are frequently linked to disruption of the intestinal epithelial barrier and gut dysbiosis, which may drive their initiation and progression [12].

Lipopolysaccharide (LPS), an outer-membrane constituent of Gram-negative bacteria, is widely used to induce inflammatory responses in intestinal cell models. LPS-recognition by Toll-like receptor 4 (TLR4) activates downstream nuclear factor-kappa B (NF-κB) and mitogen-activated protein kinase (MAPK) pathways, triggering increased production of inflammatory mediators and impairment of epithelial barrier integrity [13, 14]. Inflammation induced under these conditions may suppress the expression of barrier-associated genes and disrupt intestinal serotonergic balance by altering serotonin synthesis and reuptake [15, 16]. Because serotonin contributes to intestinal motility, mucosal immune regulation, and epithelial barrier maintenance [17], evaluating pro-inflammatory mediators, barrier-associated factors, and serotonin-related genes in combination provides a more comprehensive framework for assessing the protective activity of probiotics against intestinal inflammatory stress.

Probiotics are viable microorganisms that provide beneficial effects to the host when administered in sufficient quantities [18, 19]. Their health-promoting activities include supporting the recovery of microbial homeostasis, modulating excessive inflammatory reactions, and reinforcing the intestinal epithelial barrier. *Leuconostoc mesenteroides* is an obligately heterofermentative lactic acid bacterium that predominates during the early and intermediate phases of kimchi fermentation. Various *L. mesenteroides* strains have attracted attention as potential probiotics due to their ability to alleviate intestinal inflammation and support mucosal barrier integrity [20, 21]. In particular, the kimchi-derived strain *L. mesenteroides* H40 (H40) has demonstrated functional potential in previous cell- and animal-based studies, including neuroprotective activity and improvement of cognitive deficits [22–24].

Despite these findings, the influence of H40 on inflammatory signaling, epithelial barrier-associated factors, and serotonin-related markers in LPS-challenged intestinal epithelial cells remains incompletely characterized. Therefore, the present study evaluated the anti-inflammatory activity of H40 and examined its effects on barrier- and serotonergic homeostasis-related markers using LPS-stimulated HT-29 cells.

## Materials and Methods

### Bacterial Strains and Preparation

Kimchi-derived *L. mesenteroides* (H40) was evaluated in comparison with *Lacticaseibacillus rhamnosus* GG (LGG; Korean Collection for Type Cultures, KCTC, Republic of Korea). LGG was included as a reference probiotic strain because of its well-characterized probiotic properties and documented interactions with intestinal epithelial cells [25]. Each strain was cultured in De Man, Rogosa, and Sharpe (MRS) broth (Difco Laboratories, USA) for 18 h at 37°C. The cultures were subsequently centrifuged at 14,240 × g for 5 min at 4°C to recover the bacterial cells. After two washes with phosphate-buffered saline (PBS; HyClone Laboratories, USA), the cell pellets were resuspended in the appropriate cell culture medium and adjusted to 8 log CFU/mL.

### Cell Culture Conditions

HT-29 human colorectal adenocarcinoma cells (KCLB 30038) were purchased from the Korean Cell Line Bank (Republic of Korea). The cells were routinely maintained in Roswell Park Memorial Institute (RPMI) medium (HyClone Laboratories) supplemented with 10% fetal bovine serum (FBS; Life Technologies, USA) and 1% penicillin–streptomycin (HyClone Laboratories). Cell cultures were incubated in a humidified atmosphere with 5% CO_2_ at 37°C and subcultured at approximately 80% confluence.

### Cell Viability Assay

Cell viability was assessed using the 3-(4,5-dimethylthiazol-2-yl)-2,5-diphenyltetrazolium bromide (MTT) assay based on the method described by Kim *et al*. [26] to examine the potential cytotoxicity of the LAB strains toward HT-29 cells. The cells were seeded in 96-well plates at a density of 1 × 10^5^ cells/well and allowed to adhere for 24 h. Following treatment with each LAB strain for an additional 24 h, the cells were washed twice with PBS and incubated with MTT solution (0.5 mg/mL; Thermo Fisher Scientific, USA) for 2 h. The culture medium was then discarded, and the resulting formazan crystals were dissolved in dimethyl sulfoxide. Absorbance was recorded at 570 nm using a Multiskan GO microplate reader (Thermo Fisher Scientific). Cell viability was expressed as a percentage relative to the untreated control and calculated using the following equation:

$$\text{Cell viability (\%) =}\frac{ODsample}{ODcontrol}\times 100$$

where *ODsample* and *ODcontrol* denote the absorbance values of the LAB-treated and untreated control groups, respectively.

### Nitric Oxide (NO) Production Assay

The ability of the lactic acid bacteria (LAB) to suppress sodium nitroprusside (SNP)-induced NO production was assessed using the Griess assay. Because HT-29 cells exhibit a relatively weak NO response to LPS stimulation, SNP was used as an exogenous NO donor to directly induce nitrosative stress [27, 28]. HT-29 cells were seeded in 96-well plates at a density of 1 × 10^5^ cells/well and allowed to adhere for 24 h. The cells were subsequently preincubated with each LAB strain for 2 h and then incubated with 1 mM SNP for an additional 22 h. Following treatment, aliquots of the culture supernatants were combined with an equal volume of Griess reagent and incubated in the dark at 25°C for 15 min. Absorbance was recorded at 540 nm, and nitrite concentrations, used as an indicator of NO production, were calculated from a sodium nitrite (NaNO_2_) standard curve.

### Quantitative Real-Time Polymerase Chain Reaction (qRT-PCR) Analysis

qRT-PCR was performed based on the procedure described by Park *et al*. [14] to assess the expression of inflammation-, epithelial barrier-, and serotonin-associated genes in HT-29 cells. The cells were plated in six-well plates at a density of 1 × 10^6^ cells/well and cultured for 3 d to obtain a confluent monolayer. Each monolayer was exposed to the LAB strains for 2 h, followed by treatment with 1 μg/mL LPS for an additional 22 h.

Total RNA was extracted using TRIzol reagent (Thermo Fisher Scientific), and first-strand complementary DNA (cDNA) was subsequently synthesized using a cDNA synthesis kit (SensiFAST; Meridian Bioscience, USA). The mRNA levels of pro-inflammatory mediators, including tumor necrosis factor-alpha (*TNF-α*), cyclooxygenase-2 (*COX-2*), interleukin-1 beta (*IL-1β*), *IL-6*, and *IL-8*, were determined along with those of epithelial barrier-associated genes (zonula occludens-1 (*ZO-1*), *claudin-1*, *occludin*, Junctional adhesion molecule 1 (*JAM1*), and mucin 2 (*MUC2*)) and serotonin-related genes (Tryptophan hydroxylase 1 (*TPH1*) and serotonin transporter (*SERT*)). qRT-PCR Amplification was performed using PCR Master Mix and a QuantStudio 1 real-time PCR system (Thermo Fisher Scientific).

Different thermal cycling protocols were used based on the target genes. For *TNF-α*, *COX-2*, *IL-1β*, *IL-6*, and *IL-8*, amplification consisted of an initial denaturation at 95°C for 2 min, followed by 40 cycles at 95°C for 20 s, 65°C for 20 s, and 72°C for 30 s. For *ZO-1*, *claudin-1*, *occludin*, *JAM1*, *MUC2*, *TPH1*, and *SERT*, the initial denaturation at 95°C for 2 min was followed by 40 cycles at 95°C for 20 s and 55°C for 1 min. Relative transcript levels were normalized to *β-actin* and calculated using the 2^−ΔΔCt^ method. The primer sequences used in this study are listed in Table 1.

### Enzyme-Linked Immunosorbent Assay (ELISA)

Protein levels of claudin-1, occludin, MUC2, TPH1, and SERT were determined via ELISA. Cusabio kits (China) were used for claudin-1 and occludin, whereas MUC2, TPH1, and SERT were analyzed using kits supplied by MyBioSource (USA). HT-29 cells were plated in six-well plates and maintained for 3 d until a confluent monolayer was established. The cells were then incubated with LAB strains at 1 × 10^8^ CFU/mL for 4 h before exposure to 1 μg/mL LPS for an additional 24 h. Following treatment, the cells were collected, and protein samples were prepared and analyzed in accordance with the instructions provided with each ELISA kit.

### Whole-Genome Sequencing (WGS) and Genome Analysis

WGS of H40 was performed by CJ Bioscience (Republic of Korea). The service included DNA preparation, DNA quality control, Illumina-based library construction, 150 bp paired-end sequencing, and downstream analysis using the EzBioCloud platform. Genome assembly and coding DNA sequence (CDS) annotation were conducted from the sequencing data. The draft genome sequence was deposited in the NCBI database under WGS accession number JBVPGZ000000000 and assembly accession number GCA_055687645.1, associated with BioProject PRJNA1330410 and BioSample SAMN51521798.

### Statistical Analysis

Results are presented as the mean ± standard deviation (SD) of three independent experiments. Statistical evaluations were performed using IBM SPSS Statistics 25.0 (IBM Corp., USA). Differences among groups were assessed using one-way analysis of variance, followed by Duncan’s multiple-range test. Statistical significance was set at *p* < 0.05.

## Results

### Effects of *L. mesenteroides* H40 on HT-29 Cell Viability and SNP-Induced NO Production

The cytotoxicity of H40 was first assessed in HT-29 cells using the MTT assay (Fig. 1A). Cell viability remained at approximately 85% following treatment with either LGG or H40, whereas the SNP-treated group showed a viability of 80.34%. Thus, neither bacterial strain caused substantial cytotoxicity at the concentrations used, and the same experimental conditions were applied in subsequent analyses.

The inhibitory activity of H40 against SNP-induced NO production was then examined (Fig. 1B). Basal NO production was negligible in untreated cells but increased markedly to approximately 49 μM following SNP exposure. Treatment with both LAB strains significantly suppressed this response, reducing NO levels to approximately 36 μM with LGG and 31 μM with H40. This inhibitory effect was more pronounced with H40, demonstrating that this strain exhibited superior potency against SNP-induced NO accumulation among the tested LAB strains.

### Effects of *L. mesenteroides* H40 on Pro-Inflammatory Mediator Expression

The anti-inflammatory activity of H40 was assessed by measuring the mRNA levels of *IL-1β*, *IL-6*, *IL-8*, *TNF-α*, and *COX-2* in LPS-treated HT-29 cells (Fig. 2). LPS treatment significantly upregulated the expression of all five pro-inflammatory mediators. These increases were significantly attenuated by pretreatment with either LGG or H40, with H40 generally exerting a greater suppressive effect. Specifically, H40 pretreatment attenuated the LPS-induced upregulation of *IL-1β*, *IL-6*, *IL-8*, and *COX-2*, maintaining their expression at levels comparable to those in the untreated control. *TNF-α* expression was also significantly lower in the H40-pretreated group than in the LPS-treated and LGG-pretreated groups.

### Effects of *L. mesenteroides* H40 on Intestinal Barrier-Related Gene Expression

The effects of H40 on epithelial barrier-associated gene expression were examined by measuring the mRNA levels of *ZO-1*, *claudin-1*, *occludin*, *MUC2*, and *JAM1* in LPS-treated HT-29 cells (Fig. 3). LPS treatment significantly downregulated all five genes. In contrast, H40 pretreatment attenuated these reductions, indicating that H40 helped preserve epithelial barrier-associated gene expression under inflammatory conditions. Compared with LGG, H40 exerted greater effects on *claudin-1*, *occludin*, and *MUC2* expression, with *claudin-1* expression remaining comparable to that in the untreated control.

### Effects of *L. mesenteroides* H40 on Serotonin-Related Gene Expression

The effects of H40 on intestinal serotonin-related gene expression were examined by measuring the mRNA levels of *TPH1* and *SERT* in LPS-treated HT-29 cells (Fig. 4). LPS treatment significantly downregulated both genes. H40 pretreatment attenuated these reductions more effectively than LGG, with a particularly pronounced effect on *SERT* expression.

### Effects of *L. mesenteroides* H40 on Intestinal Barrier-Related Protein Expression

Claudin-1, occludin, and MUC2 protein levels were quantified using ELISA to determine whether the barrier-protective effects of H40 observed at the transcriptional level were also evident at the protein level (Fig. 5). LPS exposure decreased claudin-1 from 307.0 to 142.3 pg/mL and occludin from 549.3 to 220.1 pg/mL (Fig. 5A and 5B). H40 pretreatment partially restored these proteins to 200.3 and 395.9 pg/mL, respectively, whereas the corresponding levels in the LGG-treated group were 151.0 and 328.4 pg/mL. H40 exerted a greater protective effect than LGG on both tight junction-associated proteins. Similarly, LPS exposure reduced MUC2 levels from 13.4 to 7.1 ng/mL (Fig. 5C). In contrast, H40 pretreatment increased MUC2 concentration to 20.9 ng/mL, exceeding the levels observed in both the untreated control and LGG-treated groups.

### Effects of *L. mesenteroides* H40 on Serotonin-Related Protein Expression

TPH1 and SERT protein levels were quantified using ELISA to examine whether the transcriptional effects of H40 were also evident at the protein level in LPS-stimulated HT-29 cells (Fig. 6). Exposure to LPS decreased both proteins relative to the untreated control. However, H40 pretreatment prevented the decline in TPH1, maintaining its level near control levels (Fig. 6A). SERT concentration decreased from 105.8 to 81.5 ng/mL following LPS exposure but increased to 139.4 ng/mL in H40-pretreated cells (Fig. 6B). This value was also higher than the 116.2 ng/mL observed in the LGG-pretreated group.

### WGS and Genomic Characterization of *L. mesenteroides* H40

WGS and annotation were performed to characterize the genomic features of H40. The draft genome had a total size of 1,999,541 bp and a GC content of 37.6% (Table 2). The genome assembly consisted of 11 contigs, with an N50 value of 1,067,364 bp, and was classified as a draft assembly at the contig level. Genome annotation identified 1,969 coding sequences (CDSs), 52 tRNA genes, and 3 rRNA genes. The circular genome map (Fig. 7) shows the distribution of predicted CDSs, rRNA and tRNA genes, GC skew, and GC ratio across the assembled pseudogenome. In addition, several genes potentially associated with probiotic-related functions, including exopolysaccharide biosynthesis, cell-surface functions, antioxidant defense, tryptophan/aromatic amino acid metabolism, and B-vitamin metabolism, were identified, with their corresponding locus tags and predicted functions summarized in Table 3.

In addition, H40 exhibited resistance to kanamycin among the eight antibiotics tested, including ampicillin, gentamicin, kanamycin, streptomycin, erythromycin, clindamycin, tetracycline, and chloramphenicol (data not shown). Genomic analysis revealed that the gene encoding aminoglycoside 6’-N-acetyltransferase was located in the chromosomal region and was not associated with known mobile genetic elements. Therefore, the kanamycin resistance determinant in H40 is considered unlikely to be horizontally transferable, supporting the safety of H40 with respect to antibiotic resistance.

## Discussion

NO functions as an important signaling molecule in numerous intracellular and extracellular processes. However, excessive accumulation of NO under pathological conditions can promote intestinal inflammation, epithelial damage, and nitrosative stress [29, 30]. During inflammatory responses, stimuli such as LPS induce the expression of inflammatory mediators, including inducible nitric oxide synthase (iNOS). Increased iNOS activity catalyzes the conversion of L-arginine to NO and L-citrulline, thereby elevating cellular NO levels [31, 32]. However, HT-29 cells respond relatively weakly to LPS because they express low levels of the TLR4/MD-2 receptor complex [27]. Therefore, SNP was used as an exogenous NO donor to generate NO-mediated stress directly in HT-29 cells [28]. Under these conditions, *L. mesenteroides* H40 significantly suppressed SNP-induced NO accumulation without causing evident cytotoxicity and exerted a stronger effect than the reference strain LGG. Because cell viability was not substantially reduced, the lower NO level observed following H40 pretreatment was unlikely to result from a decrease in viable cell number. Instead, this finding suggests that H40 may alleviate SNP-induced cellular stress, protecting intestinal epithelial cells against excessive NO exposure and associated nitrosative injury. However, because SNP releases NO independently of iNOS expression, further investigation into oxidative and nitrosative stress-related pathways is required to elucidate the mechanisms underlying H40-mediated protection.

Inflammatory mediators are central to the development and progression of intestinal inflammation. Among these, IL-1β, IL-6, and TNF-α amplify pro-inflammatory signaling, whereas IL-8 facilitates the migration of immune cells toward damaged epithelial regions [33]. COX-2, an inducible enzyme responsible for prostanoid production, can further aggravate mucosal inflammation and tissue injury when excessively expressed [34]. Consequently, these molecules serve as indicators of inflammatory activation in intestinal epithelial cell systems, including HT-29 cells [35, 36]. In the present study, the substantial reduction in their expression following H40 pretreatment suggests that H40 may alleviate LPS-induced inflammatory responses in intestinal epithelial cells.

The intestinal epithelial barrier is maintained through the coordinated functions of tight junction proteins and the mucus layer [37]. ZO-1, claudin-1, occludin, and JAM1 are major tight junction-associated components that regulate paracellular permeability, whereas MUC2 is the principal gel-forming mucin that constitutes the protective intestinal mucus layer [38, 39]. Under inflammatory conditions, reduced expression of these barrier components can disrupt epithelial integrity, increase intestinal permeability, and promote mucosal injury [40, 41]. In the present study, H40 attenuated the LPS-induced downregulation of *ZO-1*, *claudin-1*, *occludin*, *JAM1*, and *MUC2* mRNA. Additionally, H40 mitigated the reduction of claudin-1 and occludin protein expression more effectively than LGG and upregulated MUC2 protein levels beyond control baseline. These findings suggest that H40 may preserve intestinal epithelial barrier integrity by maintaining tight junction- and mucus-related components under inflammatory conditions. However, functional analyses, including transepithelial electrical resistance and paracellular permeability assays, are required to confirm whether these molecular changes improve epithelial barrier function.

Intestinal serotonergic pathways contribute to epithelial regulation, fluid secretion, gastrointestinal motility, and communication along the gut–brain axis [42, 43]. TPH1 catalyzes the rate-limiting step in peripheral serotonin biosynthesis, whereas SERT removes serotonin from the extracellular space through cellular reuptake [44]. Dysregulation of either factor has been reported in inflammatory and functional gastrointestinal disorders, indicating that both are essential for preserving serotonergic balance in the intestine [44, 45]. In this study, H40 pretreatment mitigated the LPS-mediated suppression of *TPH1* and *SERT* mRNA levels. Furthermore, it preserved TPH1 protein expression near the untreated control levels and increased SERT protein levels beyond levels detected in both the untreated control and LGG-pretreated groups. These results suggest that H40 may influence serotonin-related pathways during inflammatory stress. However, serotonin content and transporter function should be measured directly to determine whether these molecular changes result in functional changes in intestinal serotonergic signaling.

Genome annotation revealed that H40 harbors genes associated with exopolysaccharide biosynthesis, cell-surface functions, antioxidant defense, tryptophan/aromatic amino acid metabolism, and B-vitamin metabolism (Table 3). Genes involved in exopolysaccharide production and cell-surface functions may contribute to bacterial cell-surface properties and interactions with intestinal epithelial cells. Indeed, the exopolysaccharide layer of lactic acid bacteria has been reported to influence biofilm formation, adhesion, and resistance to environmental stress [46]. The presence of antioxidant defense-related genes further suggests that H40 may possess the potential to withstand oxidative stress within the intestinal environment. In addition, genes associated with tryptophan and aromatic amino acid metabolism may be relevant to intestinal mucosal homeostasis and serotonin-related pathways through the production of microbial metabolites [47, 48]. Microbiota-derived tryptophan metabolites and serotonergic signaling have been implicated in gut–brain communication [49, 50]. However, the present study did not directly evaluate serotonin production, neural responses, or behavioral outcomes. The identified B-vitamin metabolism-related gene may contribute to the metabolic capacity of H40 as well as to nutritional interactions among members of the gut microbiota [51]. Genome-based analyses have suggested that intestinal microorganisms may cooperate metabolically through the exchange of B vitamins. Collectively, these genomic features may provide a potential molecular basis for the intestinal epithelial-protective effects and serotonin-related molecular changes observed in the present study. However, the presence of predicted genes alone does not confirm their active expression or physiological activity. Therefore, further transcriptomic, metabolomic, and targeted functional analyses are required to determine whether these genes and metabolic pathways directly contribute to the observed biological effects and serotonin-related changes.

## Conclusion

*L. mesenteroides* H40 exerted protective effects against inflammatory stress in HT-29 intestinal epithelial cells. H40 reduced SNP-induced NO accumulation and suppressed the LPS-induced expression of pro-inflammatory mediators, including *IL-1β*, *IL-6*, *IL-8*, *TNF-α*, and *COX-2*. Furthermore, H40 attenuated the LPS-induced downregulation of intestinal barrier-related genes and maintained or enhanced the protein levels of claudin-1, occludin, and MUC2. H40 also preserved TPH1 and increased SERT protein levels under inflammatory conditions, suggesting a potential influence on serotonin-related pathways. However, serotonin production and transporter function were not directly evaluated in the present study. Consistent with these cellular findings, genome annotation revealed that H40 harbors genes associated with exopolysaccharide biosynthesis, cell-surface functions, antioxidant defense, tryptophan/aromatic amino acid metabolism, and B-vitamin metabolism. These genomic features may provide a potential molecular basis for the epithelial-protective, anti-inflammatory, and serotonin-related molecular changes observed in this study. Collectively, the cellular and genomic results suggest that H40 contributes to intestinal homeostasis by attenuating inflammatory stress, preserving epithelial barrier-associated components, and influencing serotonin-related pathways. Consequently, H40 may represent a promising candidate for probiotic applications and the development of functional food products aimed at supporting intestinal health. However, further validation in animal models and clinical studies is required to confirm the functional effects of H40 *in vivo*.

## Figures and Tables

**Fig. 1:**
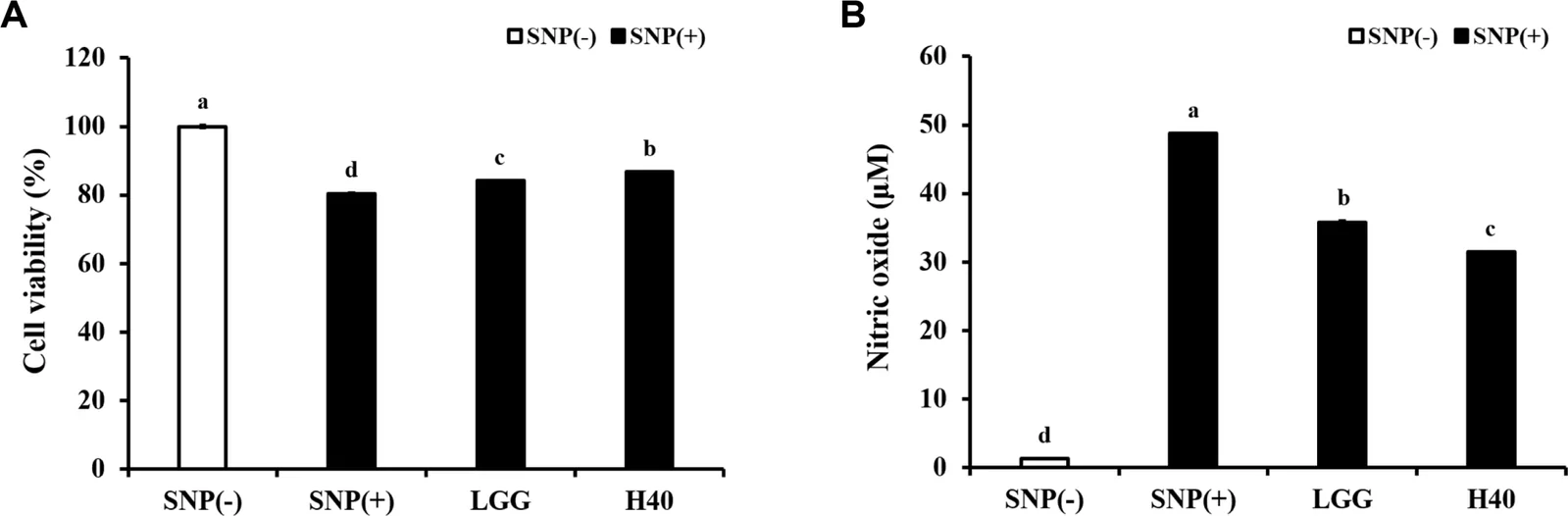
Effects of *L. mesenteroides* H40 on cell viability and NO production in SNP-treated HT-29 cells. (**A**) Cell viability and (**B**) NO production were measured in SNP-treated HT-29 cells treated with LGG or *L. mesenteroides* H40. White bars ( □ ) represent the untreated SNP(−) group, whereas black bars (■) represent the SNP (1 mM)-treated groups, including SNP(+), LGG, and H40. LGG, *L. rhamnosus* GG; H40, *L. mesenteroides* H40. Data are presented as the mean ± SD of three independent experiments. Different letters (a–d) above the bars indicate statistically significant differences (*p* < 0.05).

**Fig. 2:**
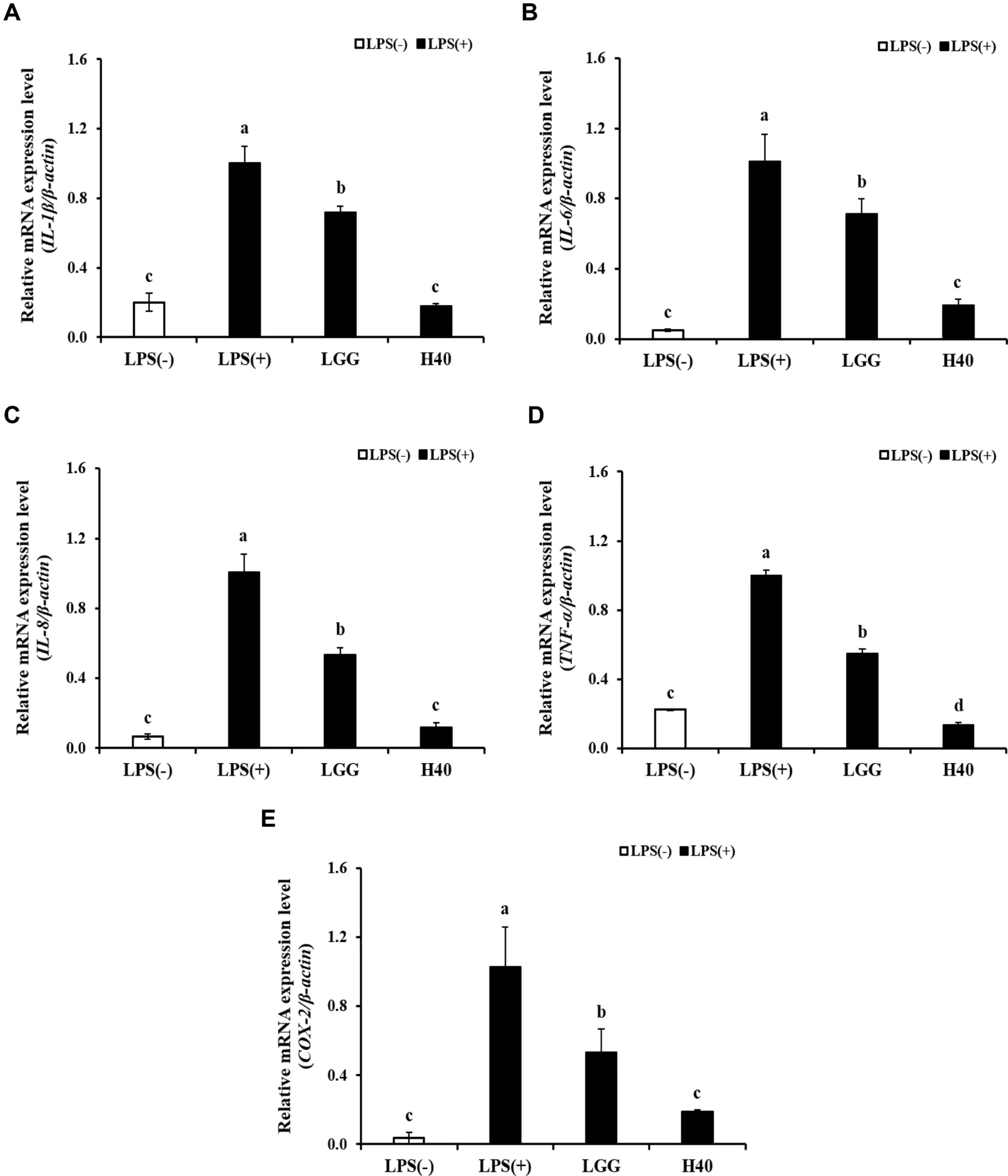
Effects of *L. mesenteroides* H40 on mRNA expression of pro-inflammatory mediators in LPS-stimulated HT-29 cells. Relative mRNA expression levels of (**A**) *IL-1β*, (**B**) *IL-6*, (**C**) *IL-8*, (**D**) *TNF-α*, and (**E**) *COX-2* were analyzed via qRT-PCR in LPS-stimulated HT-29 cells treated with LGG or *L. mesenteroides* H40. White bars ( □ ) represent the untreated LPS(−) group, whereas black bars (■) represent the LPS (1 μg/mL)-stimulated groups, including LPS(+), LGG, and H40. LGG, *L. rhamnosus* GG; H40, *L. mesenteroides* H40. Data are presented as the mean ± SD of three independent experiments. Different letters (a–d) above the bars indicate statistically significant differences (*p* < 0.05).

**Fig. 3:**
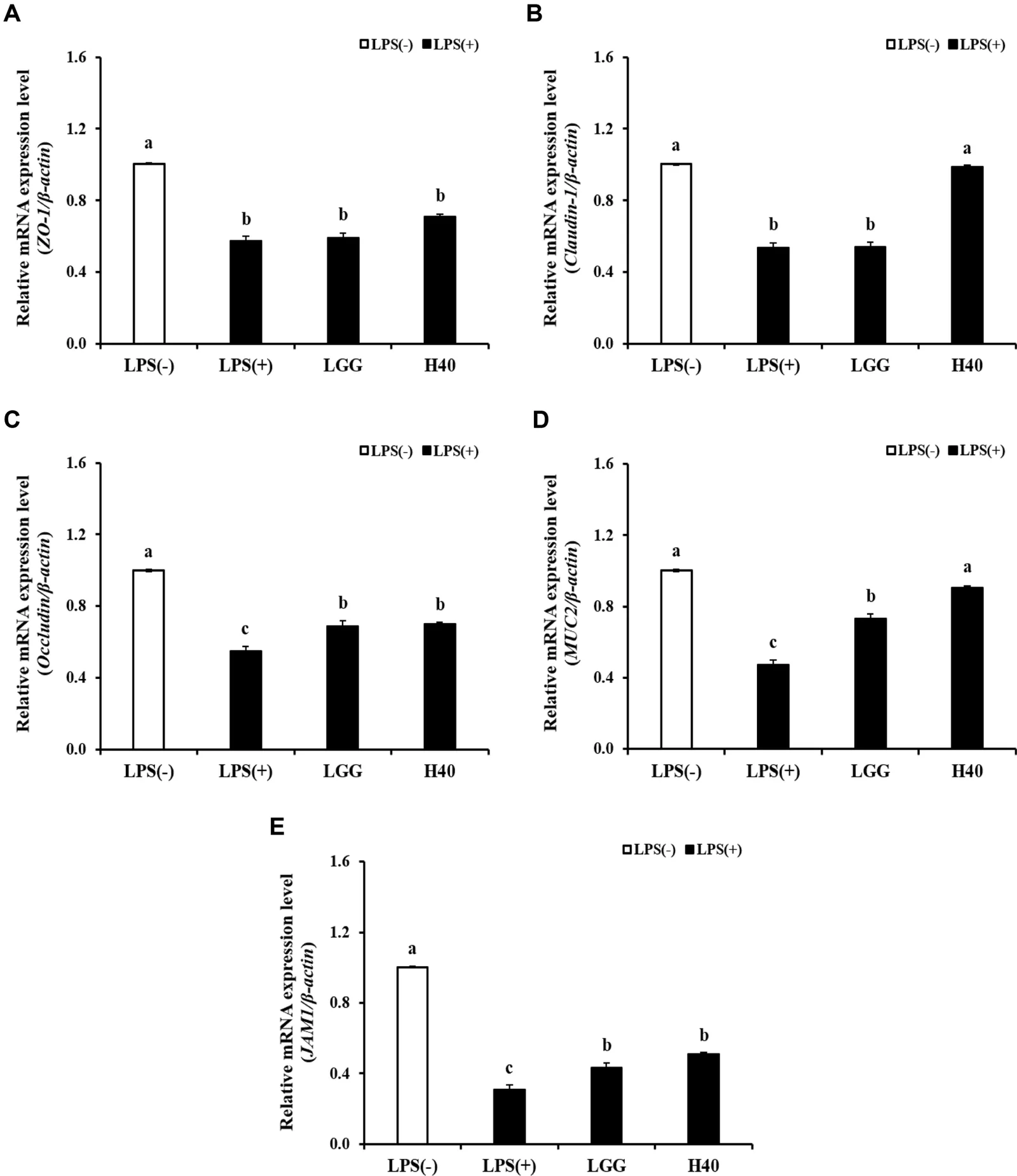
Effects of *L. mesenteroides* H40 on mRNA expression of intestinal barrier-related genes in LPS-stimulated HT-29 cells. Relative mRNA expression levels of (**A**) *ZO-1*, (**B**) *claudin-1*, (**C**) *occludin*, (**D**) MUC-2, and (**E**) *JAM1* were analyzed using qRT-PCR in LPS-stimulated HT-29 cells treated with LGG or *L. mesenteroides* H40. White bars ( □ ) represent the untreated LPS(−) group, whereas black bars (■) represent the LPS (1 μg/mL)-stimulated groups, including LPS(+), LGG, and H40. LGG, *L. rhamnosus* GG; H40, *L. mesenteroides* H40. Data are presented as the mean ± SD of three independent experiments. Different letters (a–c) above the bars indicate statistically significant differences (*p* < 0.05).

**Fig. 4:**
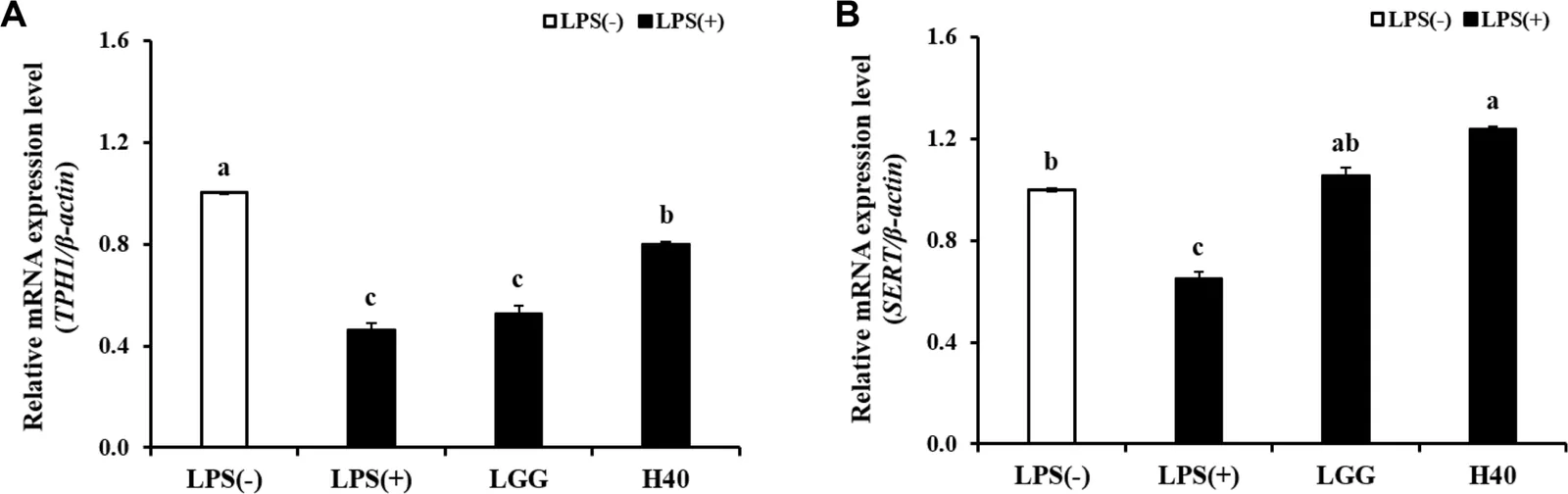
Effects of *L. mesenteroides* H40 on mRNA expression of serotonin-related genes in LPS-stimulated HT-29 cells. Relative mRNA expression levels of (**A**) *TPH1* and (**B**) *SERT* were analyzed using qRT-PCR in LPS-stimulated HT-29 cells treated with LGG or *L. mesenteroides* H40. White bars ( □ ) represent the untreated LPS(−) group, whereas black bars (■) represent the LPS (1 μg/mL)-stimulated groups, including LPS(+), LGG, and H40. LGG, *L. rhamnosus* GG; H40, *L. mesenteroides* H40. Data are presented as the mean ± SD of three independent experiments. Different letters (a–c) above the bars indicate statistically significant differences (*p* < 0.05).

**Fig. 5:**
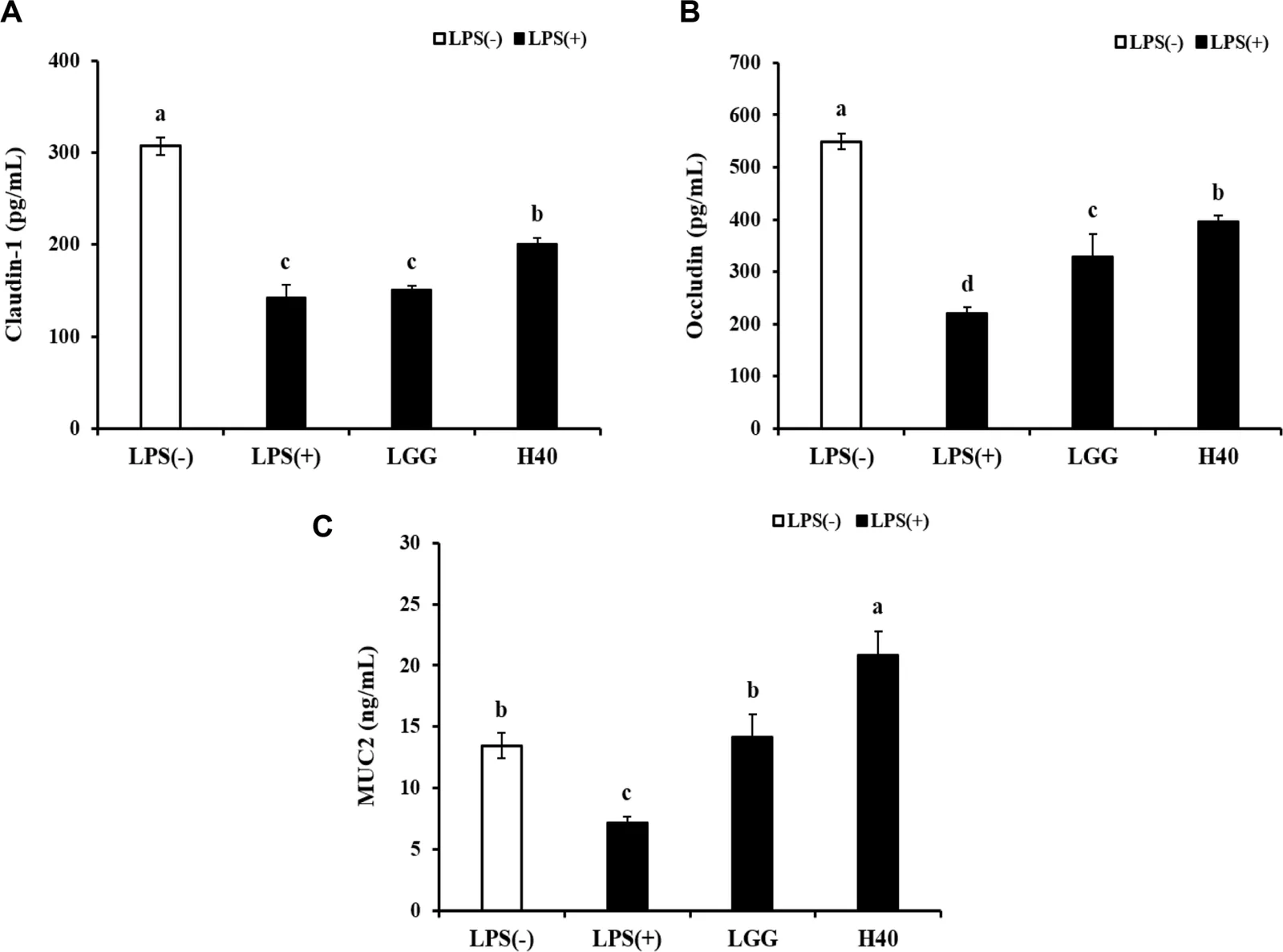
Effects of *L. mesenteroides* H40 on intestinal barrier-related protein levels in LPS-stimulated HT-29 cells. Protein levels of (**A**) claudin-1, (**B**) occludin, and (**C**) MUC2 were measured via ELISA in LPS-stimulated HT-29 cells treated with LGG or *L. mesenteroides* H40. White bars ( □ ) represent the untreated LPS(−) group, whereas black bars (■) represent the LPS (1 μg/mL)-stimulated groups, including LPS(+), LGG, and H40. LGG, *L. rhamnosus* GG; H40, *L. mesenteroides* H40. Data are presented as the mean ± SD of three independent experiments. Different letters (a–c) above the bars indicate statistically significant differences (*p* < 0.05).

**Fig. 6:**
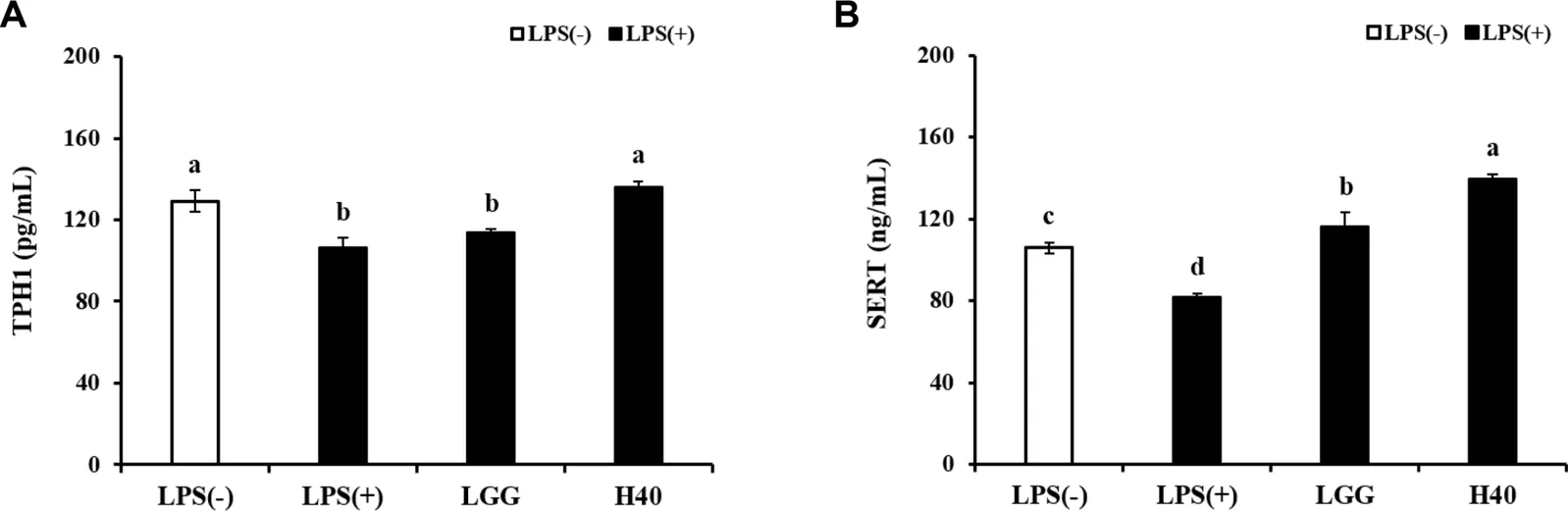
Effects of *L. mesenteroides* H40 on serotonin-related protein levels in LPS-stimulated HT-29 cells. Protein levels of (**A**) TPH1 and (**B**) SERT were measured using ELISA in LPS-stimulated HT-29 cells treated with LGG or *L. mesenteroides* H40. White bars ( □ ) represent the untreated LPS(−) group, whereas black bars (■) represent the LPS (1 μg/mL)-stimulated groups, including LPS(+), LGG, and H40. LGG, *L. rhamnosus* GG; H40, *L. mesenteroides* H40. Data are presented as the mean ± SD of three independent experiments. Different letters (a–d) above the bars indicate statistically significant differences (*p* < 0.05).

**Fig. 7:**
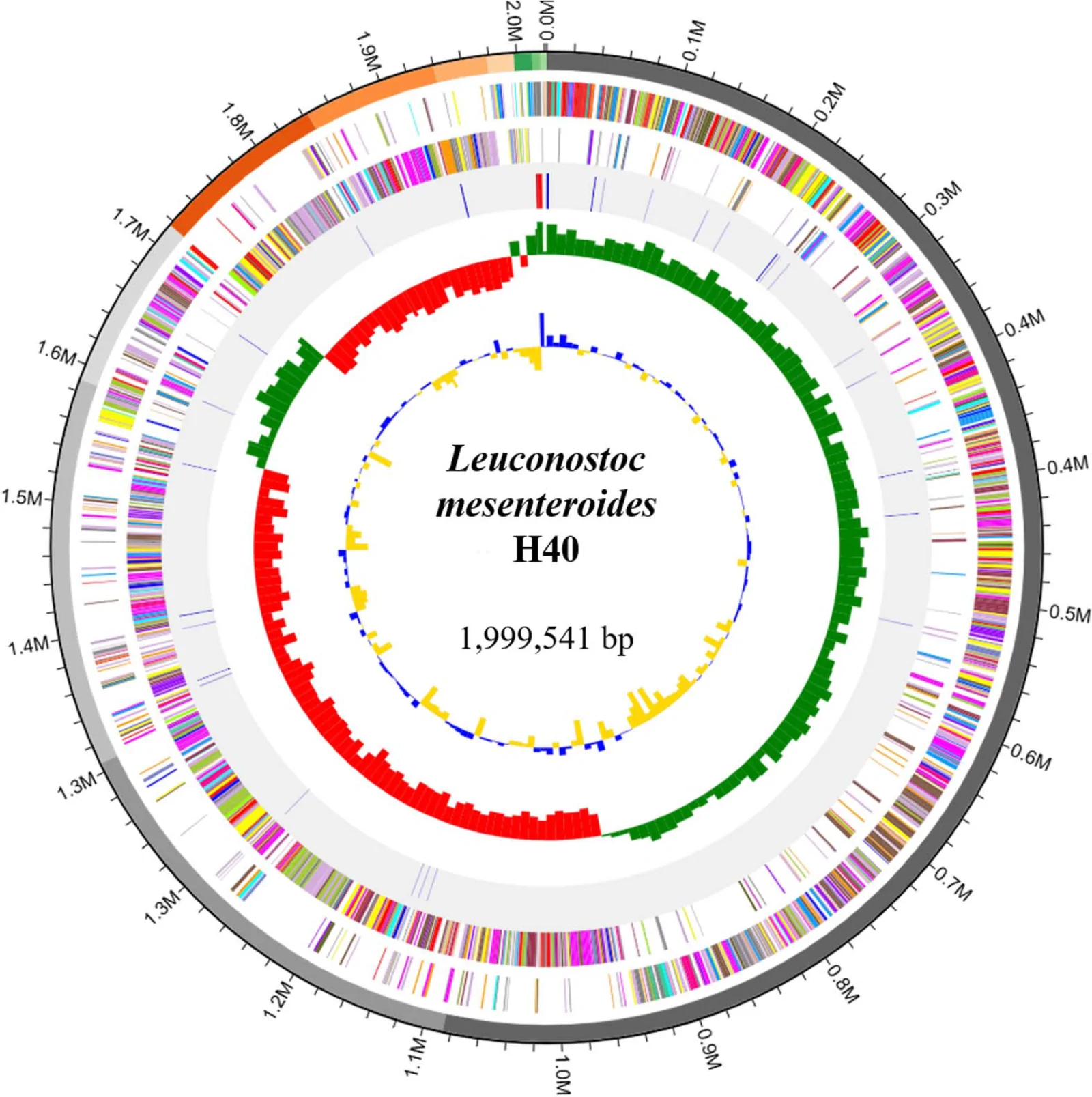
Circular representation of the draft genome of *L. mesenteroides* H40. From the outermost ring inward, the map displays assembled contigs, CDSs on the forward and reverse strands color-coded according to COG functional categories, rRNA and tRNA genes, GC skew, and GC ratio. Green and red indicate GC skew values above and below the genomic mean, respectively, whereas blue and yellow indicate GC ratio values above and below the genomic mean, respectively.

**Table 1:** Primer sequences used for qRT-PCR.

Gene		Primer sequence (5′ → 3′)
*IL-1β*	(Forward)	AGA TGA TAA GCC CAC TCT ACA G
	(Reverse)	ACA TTC AGC ACA GGA CTC TC
*IL-6*	(Forward)	CCT GAA CCT TCC AAA GAT GGC
	(Reverse)	TTC ACC AGG CAA GTC TCC TCA
*IL-8*	(Forward)	GAC GAG ACC AGG AGA AAC AGG G
	(Reverse)	AAC GGA GAA AGA AGA CAG ACT GCT
*TNF-α*	(Forward)	CTG CCC AGA CTC GGC AA
	(Reverse)	GGA GAA GGG TGA CCG ACT CA
*COX-2*	(Forward)	CAC TAC ATC CTG ACC CAC TT
	(Reverse)	ATG CTC CTG CTT GAG TAT GT
*ZO-1*	(Forward)	GAC CAA TAG CTG ATG TTG CCA GAG
	(Reverse)	TGC AGG CGA ATA ATG CCA GA
*Claudin-1*	(Forward)	GGG CAG ATC CAG TGC AAA G
	(Reverse)	GGA TGC CAA CCA CCA TCA AG
*Occludin*	(Forward)	AGT GCC ACT TTG GCA TTA TGA GA
	(Reverse)	CTT GTG GCA GCA ATT GGA AAC
*MUC2*	(Forward)	ATG CCC ACC TCC TCA AAG AC
	(Reverse)	GTA GTT TCC GTT GGA ACA GTG AA
*JAM1*	(Forward)	AGC CTA GTG CCC GAA GTG
	(Reverse)	TGT GGG GTG TAG AAG ACA AAT AA
*TPH1*	(Forward)	TGC AAA GGA GAA GAT GAG AGA ATT TAC
	(Reverse)	CTG GTT ATG CTC TTG GTG TCT TTC
*SERT*	(Forward)	TCA CAG TGC TCG GTT ACA TGG C
	(Reverse)	GAA AGT GGA CGC TGG CAT GTT C
*β-actin*	(Forward)	CTT CTA CAA TGA GCT GCG TG
	(Reverse)	TCA TGA GGT AGT CAG TCA GG

**Table 2:** General genomic characteristics of *L. mesenteroides* H40.

Attribute	*L. mesenteroides* H40
Genome size (bp)	1,999,541
GC content (%)	37.6
No. of contigs	11
N50 (bp)	1,067,364
No. of coding sequences (CDSs)	1,969
No. of tRNA genes	52
No. of rRNA genes	3
Assembly type	Incomplete (contigs)

**Table 3:** Representative genes of *L. mesenteroides* H40 potentially associated with probiotic-related functions.

Functional category	Annotated protein	EzBioCloud feature ID	Predicted function
Exopolysaccharide biosynthesis	UDP-glucose:undecaprenyl-phosphate glucose-1-phosphate transferase	H40_01190	Initiation of polysaccharide biosynthesis by transfer of glucose-1-phosphate to an undecaprenyl-phosphate carrier
	Capsular polysaccharide biosynthesis protein CapA	H40_01217	Capsular/exopolysaccharide biosynthesis
	Protein-tyrosine phosphatase, CpsB/CapC family	H40_01218	Regulation of capsular polysaccharide biosynthesis
Cell-surface functions	1,2-Diacylglycerol 3-alpha-glucosyltransferase	H40_00219	Biosynthesis of membrane glycolipids associated with Gram-positive cell-surface structures
	Diglucosyl diacylglycerol synthase (1,2-linking)	H40_00220	Glycolipid biosynthesis associated with the bacterial cell membrane and cell-surface architecture
Antioxidant defense	Peroxiredoxin (atypical 2-Cys thiol peroxidase)	H40_01467	Detoxification of peroxides, including H_2_O_2_, associated with oxidative stress defense
	Peroxiredoxin (alkyl hydroperoxide reductase)	H40_01232	Reduction of organic hydroperoxides associated with oxidative stress defense
Tryptophan/aromatic amino acid metabolism	Tryptophan synthase alpha chain	H40_00984	Formation of indole during tryptophan biosynthesis
	Tryptophan synthase beta chain	H40_00988	Biosynthesis of L-tryptophan from indole and L-serine
B-vitamin metabolism	Adenosylcobalamin/alpha-ribazole phosphatase	H40_00214	Dephosphorylation step associated with adenosylcobalamin (vitamin B12) biosynthesis
